# The Prevalence and Predicting Factors of Temporomandibular Disorders in COVID-19 Infection: A Cross-Sectional Study

**DOI:** 10.7759/cureus.28167

**Published:** 2022-08-19

**Authors:** Camille Haddad, Suzanna Maria Sayegh, Amine El Zoghbi, Ghida Lawand, Lara Nasr

**Affiliations:** 1 Prosthodontics and Occlusion Department, Faculty of Dental Medicine, Saint Joseph University of Beirut, Beirut, LBN; 2 Prosthodontics and Esthetic Dentistry Department, Faculty of Dental Medicine, Saint Joseph University of Beirut, Beirut, LBN; 3 Cranio-Facial Research Laboratory, Faculty of Dental Medicine, Saint Joseph University of Beirut, Beirut, LBN

**Keywords:** orofacial pain, covid-19 retro, temporomandibular disorders, signs and symptoms, epidemiology, covid-19

## Abstract

Introduction

During the pandemic of coronavirus disease 2019 (COVID-19), an increase in temporomandibular disorders (TMDs) was noticed in infected patients. In the present study, we aimed to assess the prevalence of TMDs during COVID-19 infection and to evaluate associated factors.

Methods

An observational cross-sectional online survey was conducted in April and May 2021 in order to estimate the prevalence of TMDs in participants who were previously infected with COVID-19. A multivariable logistic regression model was carried out to explore predicting factors of TMDs during COVID-19 infection.

Results

In total, the prevalence of TMDs during the COVID-19 infection period among participants was 41.9%. High fever episodes (adjusted odds ratio {aOR}: 3.25), gastro-esophageal reflux (aOR: 2.56), and toothache (aOR: 3.83) during COVID-19 illness were found to be positive predictors of TMDs, while vitamin D deficiency was found to be a negative predictor (aOR: 0.28).

Conclusion

Our study has highlighted a relatively high prevalence of TMDs in COVID-19-infected patients that may conclude TMDs as a possible COVID-19 symptom. Further studies are warranted to confirm the association between TMDs and COVID-19 infection and thereupon include TMDs among the known symptoms of COVID-19.

## Introduction

The coronavirus disease 2019 (COVID-19) has grown exponentially at a global level and has been declared a pandemic by the World Health Organization (WHO) [1]. Lebanon has dealt with a high incidence rate of COVID-19 and a fatality rate of 19% for hospitalized infected patients, and by July 2021, there have been 552,328 confirmed COVID-19 cases with 7,888 deaths in Lebanon reported to the WHO, which further added to the burden of the unprecedented economic crisis that has hit the country badly [2]. The rapid transmission of the severe acute respiratory syndrome coronavirus 2 (SARS-CoV-2) has emerged to mount serious challenges to the medical community leading to major impacts on the respiratory, circulatory, and digestive systems [3,4]. This outbreak has impacted the dental field as well, not only with the increased risk of exposure to the virus but also with orofacial pain including bruxism and temporomandibular disorders (TMDs) as increased symptoms related to the impact of lockdowns on mental health [5,6]. Temporomandibular disorders (TMDs) describe some painful and non-painful disorders involving the masticatory muscles, the temporomandibular joint (TMJ), and the adjacent structures [7]. The most common signs of TMDs are localized jaw pain, restricted jaw movement, and TMJ sounds during mandibular movements, and the 12 common disorders include arthralgia, myalgia, local myalgia, myofascial pain, myofascial pain with referral, four-disc displacement disorders, degenerative joint disease, subluxation, and headache attributed to TMDs [8,9]. Psychological factors are considered sufficient to provoke, worsen, and maintain these symptoms during COVID-19 infection and after it [10-12]; investigating the prevalence of these symptoms during infection is important to outline clinical strategies for patient care [12-15].

During the pandemic, an increase in TMD symptoms in infected patients was noticed in daily practice. Consequently, we aimed to assess the prevalence of TMDs during COVID-19 infection, as well as to determine some related predicting factors through the present cross-sectional survey; many characteristics and possible confounders were collected from participants including sociodemographics, fever, toothache, fatigue, and health- and stress-related characteristics, which may appear to affect the prevalence of TMDs [14-17].

## Materials and methods

This was an observational cross-sectional online survey conducted by the occlusion department of Saint Joseph University of Beirut, Faculty of Dental Medicine, located in Beirut, Lebanon, during April and May 2021 in order to evaluate the prevalence of TMDs in participants during their COVID-19 infection. The protocol of this study was approved by the Research Council and the Ethics Committee of Saint Joseph University of Beirut, Faculty of Dental Medicine, on March 24, 2021 (approval number USJ-2021-76). Data were collected anonymously with no identifying information.

Participants were eligible if they were previously infected with SARS-CoV-2 confirmed by a PCR test. Using the Epi Info™ software, version 7.2.4.0 (Centers for Disease Control and Prevention {CDC}, Atlanta, GA, USA) and considering an expected prevalence of TMDs during COVID-19 of 50%, a confidence level of 95%, and an acceptable margin of error of 9%, the minimal sample size calculated was 119 participants.

The study instrument was developed in English and Arabic languages and presented using the Google Forms platform; it comprised questions related to TMDs, clenching/grinding, anxiety, and depression and questions referring to gender, demographics, TMD- and obstructive sleep apnea-related characteristics before contracting the disease, and COVID-19-related characteristics. Before the final version was distributed, expert judgements on the importance, clarity, and intelligibility of the questionnaire’s content were considered. The main outcome variable of the study was the prevalence of TMDs during COVID-19 infection (TMDs worsened or appeared during COVID-19 infection); it was assessed based on the following: 1) worsening of previously present teeth clenching/grinding and/or jaw problem during the infection; 2) pain in the temple, face, and masticatory muscles during the infection; and 3) jaw locking during the infection.

Participants were also asked to indicate on a visual analogue scale, from 0 to 10, how much they were stressed during their COVID-19 illness; a modified Bloom’s cutoff point was used to categorize this index into three levels of stress: low (score range: 0-4), moderate (score range: 5-6), and high (score range: 7-10). Regarding the fever episode variable, it referred to having or not experiencing episodes of oral temperature higher than 38°C during the illness. The ascertainment of physician-diagnosed gastro-esophageal reflux during COVID-19 illness was self-reported as well; participants were asked to indicate if they have been told by a physician that they suffered from gastro-esophageal reflux and if they have experienced heartburn and/or upper stomach pain and/or nausea and/or stomach contents’ regurgitation during their COVID-19 infection. In addition, the variable of having vitamin D deficiency was assessed based on the following question: “In your previous blood test, and if measured, was your serum 25(OH)D level lower than 20 ng/ml (or 30 nmol/L)?”

The survey link accompanied by an invitation text was distributed online via social networking platforms (such as Facebook and WhatsApp) using a snowball-sampling technique as it appeared to be the most efficient and feasible method during the pandemic. Participants got automatically directed to the study aim and informed consent pages upon receiving and clicking the link. The allocated time for the questionnaire completion was around eight minutes.

All data were transferred into and analyzed using IBM SPSS Statistics version 26.0 for Windows (IBM Corp., Armonk, NY). All analyses were two-sided, and a p-value of < 0.05 was considered statistically significant. To summarize the categorical variables, descriptive statistics were presented as frequency/percentage. To explore determinants of the dependent variable (prevalence of TMDs during COVID-19 illness), bivariate analyses were first carried out with the independent variables using chi-square and Fisher’s exact tests accordingly, and those having a p-value of ≤ 0.2 were then included in a binary logistic regression model where adjusted odds ratio (aOR) values and their 95% confidence intervals (95% CI) were calculated. The model originally included the following variables: age groups, stress level during the infection, fever episodes during the infection, gastro-esophageal reflux during the infection, toothache during the infection, and vitamin D deficiency.

## Results

In total, 193 responses were received during the survey period, out of which 69 were excluded due to missing information regarding the main outcome of the study. The remaining 124 participants previously infected with SARS-CoV-2 were included in the analysis. The general characteristics of the study population are shown in Table 1. Seventy-five participants (60.5%) were 35-years-old and younger, and 79 (63.7%) were females.

**Table 1:** General characteristics of the study population (N = 124) TMD: temporomandibular disorder

Characteristics		Frequency (N)	Percentage (%)
Sociodemographic characteristics			
Age groups (years)	≤ 35	75	60.5
	> 35	49	39.5
Sex	Male	45	36.3
	Female	79	63.7
Marital status	Single living alone	22	17.7
	Single living with parents	46	37.1
	Married without children	11	8.9
	Married with children	45	36.3
Country of residence	Lebanon or Syria	80	80.8
	Others	19	19.2
TMD- and obstructive sleep apnea-related characteristics before COVID-19 infection			
Teeth clenching or grinding	Yes	48	38.7
	No	76	61.3
Jaw or masticatory muscle problem	Yes	58	46.8
	No	66	53.2
Obstructive sleep apnea	Yes	44	35.5
	No	80	64.5
COVID-19-related characteristics			
Stress level during the infection	Low	19	15.3
	Moderate	69	55.6
	High	36	29.0
Fatigue during the infection	Yes	49	39.5
	No	75	60.5
Worsening of teeth clenching/grinding and/or jaw problem during the infection (n = 76)	Yes	35	46.1
	No	41	53.9
Pain in the temple, face, and masticatory muscles during the infection	Yes	40	32.3
	No	84	67.7
Jaw locking during the infection	Yes	9	7.3
	No	115	92.7
TMD worsened or appeared during the infection	Yes	52	41.9
	No	72	58.1
Worsening of obstructive sleep apnea during the infection (n = 44)	Yes	25	56.8
	No	19	43.2
Fever episodes during the infection	Yes	51	41.1
	No	73	58.9
Gastro-esophageal reflux during the infection (n = 122)	Yes	42	33.9
	No	80	64.5
Loss of taste during the infection	Yes	87	70.2
	No	37	29.8
Toothache during the infection	Yes	28	77.4
	No	96	22.6
Need of oxygen supply during the infection (n = 123)	Yes	7	5.7
	No	116	94.3
Hospitalization during the infection (n = 121)	Yes	7	5.8
	No	114	94.2
Masticatory function after recovering from COVID-19 (n = 98)	Normal	33	33.7
	Altered	65	66.3

Regarding the country of residence, it was categorized based on the relative political and economic stability of the country; 19 participants were located in stable countries such as France, the United Arab Emirates, Kuwait, and the United Kingdom, whereas 80 were located in Lebanon or Syria (considered as politically and economically unstable), and 25 did not mention their country of residence.

Regarding TMD-related characteristics before contracting COVID-19, participants were asked to indicate their jaw problem, if they have any, and answers were distributed as follows: 13 participants had jaw, neck, and shoulder pain; 11 had jaw clicking; eight experienced morning jaw pain and headache; eight sustained painful episodes in the temporomandibular joint; six had pain in the temples and cheeks while chewing; four had limited mouth opening; and three mentioned getting headaches after chewing hard food. Only 29 participants out of 44 who stated having obstructive sleep apnea have indicated the level of severity of their condition: 21 have mild, six have moderate, and two have advanced obstructive sleep apnea.

The rate of TMDs (worsening of previous TMDs or new onset) during the COVID-19 infection period among participants was 41.9%. The results of the bivariate analyses taking into account the main outcome variable of the study (TMDs during COVID-19 infection) are shown in Table 2. Variables such as stress during the infection and having vitamin D deficiency showed a p-value of less than or equal to 0.2 in the bivariate analysis. In addition, variables such as age groups, experiencing high fever episodes, gastro-esophageal reflux, and toothache during the infection showed statistically significant associations with TMDs during COVID-19 infection in the bivariate analysis (Table 2).

**Table 2:** Results of bivariate analyses for associations between TMDs during COVID-19 infection and selected predicting variables *p < 0.2; **p < 0.05; TMDs: temporomandibular disorders

		TMDs during COVID-19 infection		
Variables		No	Yes	p-value
Age groups (years)	≤ 35	49 (65.3%)	26 (34.7%)	0.042**
	> 35	23 (46.9%)	26 (53.1%)	
Sex	Male	28 (62.2%)	17 (37.8%)	0.479
	Female	44 (55.7%)	35 (44.3%)	
Marital status	Single living alone	13 (59.1%)	9 (40.9%)	0.226
	Single living with parents	30 (65.2%)	16 (34.8%)	
	Married without children	8 (72.7%)	3 (27.3%)	
	Married with children	21 (46.7%)	24 (53.3%)	
Country of residence	Lebanon or Syria	44 (55.0%)	36 (45.0%)	0.819
	Others	11 (57.9%)	8 (42.1%)	
Stress level during the infection	Low	12 (63.2%)	7 (36.8%)	0.060*
	Moderate	45 (65.2%)	24 (34.8%)	
	High	15 (41.7%)	21 (58.3%)	
Fatigue during the infection	Yes	27 (55.1%)	22 (44.9%)	0.589
	No	45 (60.0%)	30 (40.0%)	
Obstructive sleep apnea	Yes	26 (59.1%)	18 (40.9%)	0.864
	No	46 (57.5%)	34 (42.5%)	
Fever episodes during the infection	Yes	22 (43.1%)	29 (56.9%)	0.005**
	No	50 (68.5%)	23 (31.5%)	
Gastro-esophageal reflux during the infection	Yes	18 (42.9%)	24 (57.1%)	0.009**
	No	54 (67.5%)	26 (32.5%)	
Loss of taste during the infection	Yes	50 (57.5%)	37 (42.5%)	0.837
	No	22 (59.5%)	15 (40.5%)	
Toothache during the infection	Yes	8 (28.6%)	20 (71.4%)	< 0.001**
	No	64 (66.7%)	32 (33.3%)	
Having vitamin D deficiency	Yes	21 (67.7%)	10 (32.3%)	0.151*
	No	46 (52.9%)	41 (47.1%)	

The results of the multivariable logistic regression analysis showing determinants of TMDs in COVID-19 infection are presented in Table 3. Those who experienced fever episodes during COVID-19 infection were more likely to experience worsening of previous TMDs or new onset of TMDs compared to those who did not experience high fever episodes (aOR: 3.25, 95% CI: 1.38-7.63, p = 0.007). In addition, participants who experienced gastro-esophageal reflux during illness were more likely to show signs of TMDs compared to those who did not (aOR: 2.56, 95% CI: 1.01-6.48, p = 0.039). Similarly, participants who have experienced toothache during COVID-19 infection were almost four times more likely to experience TMDs compared to those who did not have any toothache (aOR: 3.83, 95% CI: 1.32-11.12, p = 0.013). Differently, we found that vitamin D deficiency was a negative predictor of TMDs in COVID-19-infected patients (aOR: 0.28, 95% CI: 0.098-0.83, p = 0.021) (Table 3).

**Table 3:** Logistic regression model showing predicting factors of TMDs in COVID-19 infection (N = 116) In the backward elimination logistic regression to predict temporomandibular disorders (TMDs) in COVID-19 infection, variables entered in step 1 were age groups, stress level during the infection, fever episodes during the infection, gastro-esophageal reflux during the infection, toothache during the infection, and having vitamin D deficiency. Nagelkerke R-square = 0.275; Hosmer-Lemeshow: p = 0.994; *P < 0.05; †aOR = adjusted odds ratio; ‡95% CI = 95% confidence interval

Variable		aOR†	95% CI‡	p-value
Fever episodes during the infection	No (reference)	-	-	
	Yes	3.25	1.38-7.63	0.007*
Gastro-esophageal reflux during the infection	No (reference)	-	-	
	Yes	2.56	1.01-6.48	0.039*
Toothache during the infection	No (reference)	-	-	
	Yes	3.83	1.32-11.12	0.013*
Vitamin D deficiency	No (reference)	-	-	
	Yes	0.28	0.098-0.83	0.021*

## Discussion

The COVID-19 pandemic, multiple lockdowns, and home confinement measures have had many negative and harmful effects, not only on the worldwide economy but also on mental and physical health. Several studies have concluded worsening or aggravation of TMD symptoms due to the pandemic as a result of stress and psychosocial impacts [5,6,10-14,18-22], but to our knowledge, none has yet investigated TMDs as a possible symptom of COVID-19 infection; therefore, we chose to conduct a survey to assess the prevalence of TMD onset or aggravation of previous symptoms and determine possible predictors affecting this prevalence.

In the current study, the prevalence of TMDs during disease contraction was 41.9%; this relatively high rate could be explained by several factors. The fact that most of the respondents (80%) come from politically and economically unstable countries (Lebanon and Syria) where the management of the pandemic might have been less efficient adds to the stress factor that is known to influence the prevalence of TMDs. This would also justify the high prevalence of TMD- and obstructive sleep apnea-related characteristics before COVID-19 infection. On a very important note, considering that many of the respondents stated that they suffered from previous jaw problems before contracting COVID-19 and that nearly half of them (46.1%) said that the symptoms were aggravated when they were infected allows to state that COVID-19 contraction does not necessarily but may, as mentioned in other studies [18,23], aggravate jaw problems and myofascial pain. In addition, an interesting unusual study conducted by Kardeş and Kardeş [24] that evaluated the trends in Google searches as an indication of public interest in bruxism and its symptoms during the COVID-19 pandemic both worldwide and in the United States showed that the relative search volume for these words was significantly increased during the pandemic. Concerning the pain in the temples, face, and masticatory muscles during mouth opening or while chewing during their infection period mentioned by some respondents, it can be justified by the general fatigue and muscle pain known to be symptoms of COVID-19, and that therefore got mirrored in the head and neck regions. It can therefore be deduced that pain in temporomandibular joints and related structures, as well as teeth grinding, can be intensified by COVID-19 infection [11,12,19]. The previous statement can also be explained by the elevated levels of stress and anxiety induced by the pandemic [5,18,21,22]. The study conducted by Emodi-Perlman et al. [10] confirmed that the aggravation of the psycho-emotional status caused by the pandemic can result in bruxism and TMD symptom exacerbation and hence give rise to orofacial pain. Additionally, according to other studies, it is psychological factors associated with the pandemic that may effectively lead to a greater risk of developing, worsening, and perpetuating TMDs [5,13,19]. Moreover, aggravation of TMD pain was classified as a result of the pandemic’s adverse effects on subjects’ psycho-emotional status (stress, anxiety, and depression) in general rather than symptoms directly related to COVID-19 contraction [12,20]. However, our results showed that TMDs newly appeared in some infected patients, while these disorders were never present before the coronavirus infection; that leads us to think that TMDs might be a symptom of COVID-19 infection.

Our multivariable analysis showed that those who experienced fever episodes during their infection were more than three times more likely to sustain TMDs. This aligns with the results of a few papers that reported the presence of both jaw pain and fever in some patients [16,25,26]. Additionally, gastro-esophageal reflux during the infection was a positive predictor of TMDs as well, which corroborates with results found in several studies that assessed the association between gastro-esophageal reflux disease (GERD) and TMDs [27,28]. Two case-control studies conducted by Li et al. [27] and Gharaibeh et al. [28] have highlighted the significant increase of temporomandibular disorders in patients suffering from symptomatic GERD. Li and coworkers [27] found that having gastro-esophageal reflux increases TMDs by an odds ratio of 2.74, a result very similar to the odds ratio found in our study (aOR = 2.56). Furthermore, toothache turned out to be a risk factor for TMDs in coronavirus-infected patients; this can be explained by the possible non-odontogenic dental pain as a result of myofascial pain and TMDs [17]. In contrast, our results suggest that vitamin D deficiency is a negative predictor of TMDs in COVID-19 infection. That comes in contradiction with the results of several studies that found that vitamin D deficiency is positively related to TMDs [29,30].

To our knowledge, this is the first study to assess the prevalence of TMDs in COVID-19-infected patients that allowed reporting an additional symptom of COVID-19 while exploring potential predictors of this prevalence due to the fact that the sample size was large enough to carry out a multivariable regression model. In addition, the fact that the survey was developed in English and Arabic languages may have reduced the selection bias by breaking possible language barriers and therefore allowing optimal participation in the survey. However, using the non-probability snowball-sampling method and the online-surveying method, which is restricted to computerized and internet-friendly users, may have generated a selection bias and may consequently have compromised the generalizability of our results.

## Conclusions

Our study has highlighted a relatively high prevalence of TMDs in COVID-19-infected patients that may conclude TMDs as a possible COVID-19 symptom. Furthermore, since high fever episodes, toothache, and gastro-esophageal reflux were found to be risk factors associated with TMDs during COVID-19 infection, treating them would result in risk reduction of TMDs’ new onset or worsening of previous TMDs. However, further studies are warranted to confirm the association between TMDs and COVID-19 infection and thereupon include TMDs among the known symptoms of COVID-19.
